# Barbituric Acid Based Fluorogens: Synthesis, Aggregation-Induced Emission, and Protein Fibril Detection

**DOI:** 10.3390/molecules25010032

**Published:** 2019-12-20

**Authors:** Siyang Ding, Bicheng Yao, Louis Schobben, Yuning Hong

**Affiliations:** Department of Chemistry and Physics, La Trobe Institute for Molecular Science, La Trobe University, Melbourne, VIC 3086, Australia; Siyang.Ding@latrobe.edu.au (S.D.); b.yao@latrobe.edu.au (B.Y.); 19341287@students.latrobe.edu.au (L.S.)

**Keywords:** barbituric acid, aggregation-induced emission, solvatochromism, protein fibril detection

## Abstract

Fluorescent dyes, especially those emitting in the long wavelength region, are excellent candidates in the area of bioassay and bioimaging. In this work, we report a series of simple organic fluorescent dyes consisting of electron-donating aniline groups and electron-withdrawing barbituric acid groups. These dyes are very easy to construct while emitting strongly in the red region in their solid state. The photophysical properties of these dyes, such as solvatochromism and aggregation-induced emission, are systematically characterized. Afterward, the structure–property relationships of these barbituric acid based fluorogens are discussed. Finally, we demonstrate their potential applications for protein amyloid fibril detection.

## 1. Introduction

In the past decades, fluorescence technique had been widely applied in many areas, e.g., bioimaging [1,2], clinical diagnosis [3], and optoelectronics [4]. As the fundamental element of fluorescence technique, fluorescent materials have drawn considerable research attention. Organic fluorescent dye, with the advantages of high quantum efficiency, tunable emission color, easy-approachable post-functionalization, and low toxicity, plays an important role among all kinds of fluorescent materials. However, conventional organic fluorescent dyes usually emit bright fluorescence in the dilute solutions but suffer from the notorious effect of aggregation-caused quenching (ACQ) in the condensed phase [5]. When dispersed in aqueous media or fabricated into solid film, the emission of conventional organic fluorescent dyes is usually weakened or even quenched, which greatly reduces their performance as bio-probes or in optoelectronic devices manufacture. In 2001, an abnormal photophysical phenomenon was observed and reported by the Tang’s group: hexaphenylsilole is non-emissive when molecularly dissolved but can be induced to emit intensely upon aggregation [6]. Since the emission was induced by aggregation, this novel phenomenon was defined as ‘‘aggregation-induced emission (AIE)’’. This finding provides an efficient method to overcome the ACQ effect and greatly broaden the application of organic fluorescent dyes in the real world [7,8,9,10,11].

Up to now, numerous fluorophore cores with AIE properties have been reported, e.g., tetraphenylethene (TPE) [12], distyrylanthracene (DSA) [13], and tetraphenylpyrazine (TPP) [14]. However, most of the AIE-active molecules derived from the abovementioned cores emit blue or green fluorescence, while very few of them show long-wavelength emission. In order to obtain acceptable penetration depth and avoid the auto-fluorescence of a biological matter, long-wavelength fluorescent dyes are usually in high demand [15]. The most commonly used strategy to construct organic fluorescent dyes with both AIE features and long-wavelength emission is to incorporate AIE fluorophore core into flat and extended conjugated frameworks or strong donor–acceptor skeletons [16,17,18,19,20]. Nevertheless, this strategy normally requires tedious multistep synthesis and usually gives AIE molecules with bulky structures. Therefore, it is still of great value to discover AIE fluorescent dyes with long-wavelength emission and simple structure. 

Barbituric acid (BA) is an organic compound based on a pyrimidine heterocyclic skeleton that was initially used as a central nervous system depressant. Using the Knoevenagel condensation reaction, BA can form a large variety of barbiturate derivatives which can be used as organic fluorescent dyes. In 2013, Shukla et al. reported a series of novel BA-based compounds [21]. These compounds displayed good photoluminescent properties, high molar extinction coefficient, and large Stokes shift. In 2015, Cui et al. reported a series of fluorescent probes with BA acceptors for in vivo detection of amyloid plaques [22]. Due to the planar structure and strong electron-withdrawing ability of BA moieties, all these probes displayed long-emission wavelengths and large Stokes shifts, as well as high affinities for amyloid β-peptide aggregates. Additionally, two structurally simple and red-light-emitting fluorescent dyes bearing BA acceptor were reported by Li et al. in 2016 [23]. Both of the molecules show typical characteristics of AIE and mechanochromism. Inspired by these works, we designed and synthesized a series of BA-based fluorogens and investigated their structure–property relationships, which are of vital importance for guiding the design of red-emission AIE fluorescent dyes. Subsequently, photophysical properties, e.g., AIE and solvatochromism, were systematically studied, followed by demonstration of their potential application in differentiation of different types of protein aggregates.

## 2. Results and Discussion

### 2.1. Dye Design and Synthesis

**Scheme 1:**
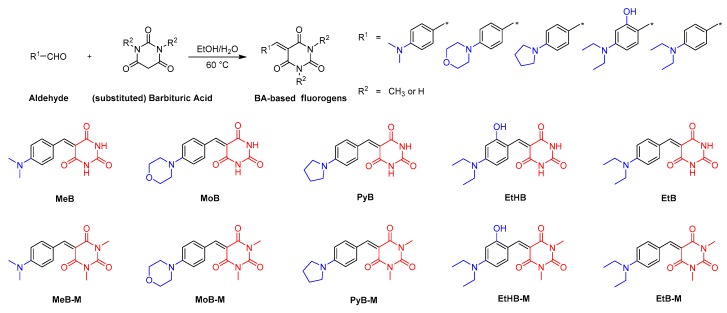
Synthetic routes to BA-based fluorogens.

The synthetic routes and chemical structures of target BA-based fluorescent dyes are shown in Scheme 1. Ten dyes were one-step synthesized by the Knoevenagel condensation of 4-benzaldehyde derivatives and barbituric acid or 1,3-dimethyl barbituric acid. Detailed information of the synthesis procedure can be found in the Materials and Methods section. Among these dyes, while the synthesis of MeB, MeB-M, EtB, EtB-M, EtHB, and MoB has already been reported by previously works [21,22,23,24,25], PyB, PyB-M, EtHB-M, and MoB-M are novel compounds. For the purpose of studying structure–property relationships, substituent groups with different electron-donating ability, e.g., *N,N*-dimethylaniline, *N,N*-diethylaniline, *N*-phenylpyrrolidine, and *N*-phenylmorpholine, were linked with BA groups via a C=C double bond. Moreover, 1,3-dimethyl BA group was introduced as an electron-withdrawing moiety to study the effect of intermolecular interactions on their photophysical properties. Furthermore, dye EtHB and EtHB-M with hydroxy group were designed to improve their water solubility. Finally, all products were fully characterized by ^1^HNMR, ^13^C NMR, and high-resolution mass spectra (HRMS), which gave satisfactory results corresponding to their chemical structures. The spectral characterization of the previously reported compounds is also consistent with the values reported in the literature [21,22,23,24,25].

### 2.2. Structure–Property Relationships

#### 2.2.1. Absorption and Emission Spectra

**Figure 1:**
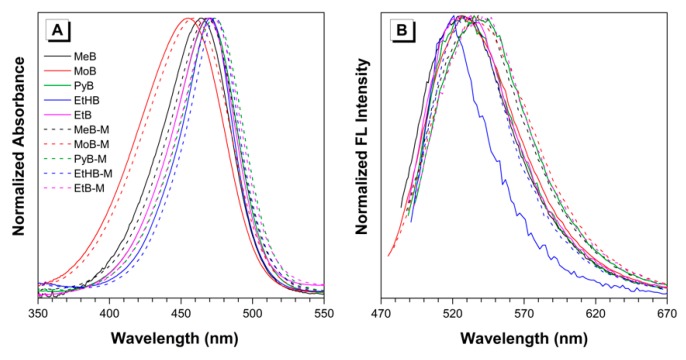
Normalized (**A**) absorption and (**B**) emission spectra of the BA-based dyes in DMSO. Concentration = 10 μM. Excitation wavelength: 464 (MeB), 468 (MeB-M), 455 (MoB), 459 (MoB-M), 469 (PyB), 473 (PyB-M), 470 (EtHB), 474 (EtHB-M), 469 (EtB), and 473 nm (EtB-M).

With the desired fluorescent dyes in hand, UV absorption and fluorescence emission spectra were first measured in DMSO. As shown in Figure 1, absorption and emission profiles of all the dyes were analyzed by normalized absorption spectra. It is obvious that all the absorption spectra have similar profiles and the maximum absorbance wavelengths all locate in the range of 450–480 nm. Specific values of the maximum absorbance wavelength were summarized in Table 1. As we can see, dyes PyB, EtB, and EtHB reached maximal absorbance at very similar wavelengths (469, 469, and 471 nm, respectively), while dye MeB exhibited a slight blue-shift at about 464 nm. Due to the twisted structure and weak electron-donating ability of morpholine, MoB showed a distinct blue-shift at about 455 nm. Furthermore, all the dyes with a methylated BA group exhibited red-shifts of 3–4 nm when compared with the maximum absorbance of their non-substituted analogues. Subsequently, the emission spectra of the BA-based luminogens were measured as excited at their recorded absorption maxima. As shown in Figure 1B and Table 1, dyes MeB, MoB, PyB, and EtB exhibited similar emission maxima at 526, 527, 527, and 532 nm, respectively, in dilute DMSO solution. However, dye EtHB showed a slight blue-shift with the maximum at 521 nm. In addition, emission spectra of the methylated dyes exhibited ~10 nm red-shifts when compared with the ones of their analogues, owing to the extension of conjugation through the methyl groups. In order to get more understanding of the photophysical properties of solid samples, fluorescence emission and quantum yields of solid samples were measured. According to the data in Table 1, all the BA-based dyes emitted orange or red (589~656 nm) light with relatively high quantum yields (up to 32.8%); as an exception, EtHB and EtHB-M showed weak emission due to the introduction of hydroxy group. 

#### 2.2.2. Effect of Solvent Polarity

**Figure 2:**
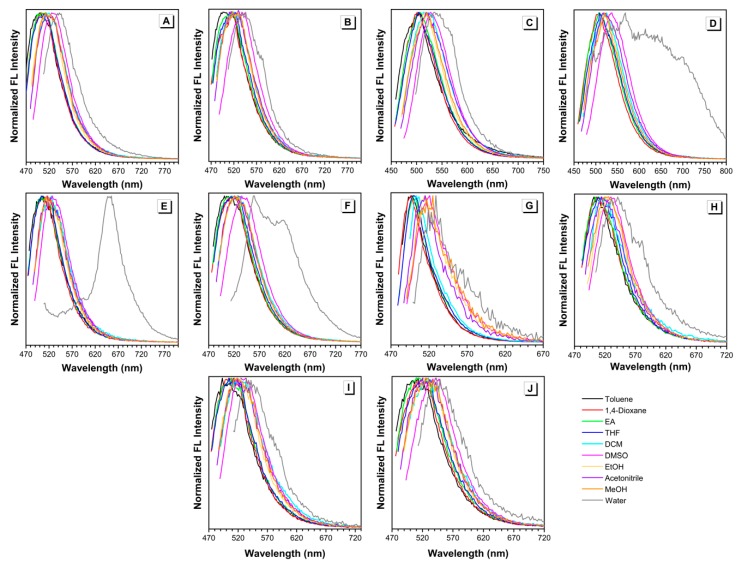
Normalized fluorescence emission spectra of (**A**) MeB, (**B**) MeB-M, (**C**) MoB, (**D**) MoB-M, (**E**) PyB, (**F**) PyB-M, (**G**) EtHB, (**H**) EtHB-M, (**I**) EtB, and (**J**) EtB-M in different solvents. Concentration = 10 µM. *λ*_ex_ = 438~494 nm.

The sensitivity of these BA-based luminogens to solvent polarity was further investigated. UV-vis absorption spectra of all the dyes in different solvents were presented in Figures S1 and S2. Using the wavelength of maximal absorption as the excitation wavelength, emission spectra of all the dyes in different solvents can be obtained as well (Figure 2 and Figure S3). 

The absorption maxima of dyes MeB and MeB-M, obtained from the normalized absorption spectra in different solvents, ranged from 448 to 488 nm and 450 to 488 nm, respectively (Figure S2A,B). It was noteworthy that the absorption spectra of MeB-M in water and 1,4-dioxane were double-peaked and remarkedly weaker than those in other solvents. According to our previous work, this phenomenon can be ascribed to the poor solubility of MeB-M in the corresponding solvents [26]. Those two peaks at short wavelength region indicated the formation of large aggregates. The corresponding fluorescence emission spectra of dyes MeB and MeB-M in different solvents were shown in Figure S3A,B. The strongest emission was observed in DMSO, while the lowest emission occurred in water. As shown in Figure 2A,B, the normalized emission spectra indicated that emission maxima of dyes MeB and MeB-M in different solvents ranged from 500 to 542 nm and 496 to 546 nm, respectively.

The absorption maxima of dyes MoB and MoB-M in different solvents ranged from 438 to 478 nm and 440 to 475 nm, respectively (Figure S2C,D). Similar phenomenon can be observed in water due to the solubility factor. In terms of fluorescence emission spectra, the results of dyes MoB and MoB-M in different solvents were shown in Figure S3C,D, respectively. For both dye MoB and dye MoB-M, strongest emission can be observed in DMSO, while the lowest emission occurred in water, which is similar to the spectra of dyes MeB and MeB-M. The emission maxima of MoB and MoB-M in different solvents were recorded to range from 502 to 535 nm and 507 to 568 nm (Figure 2C,D). Additionally, an obvious shoulder peak appeared in the spectrum of dye MoB-M in water. Specifically, the maximum of this shoulder peak was located at 614 nm, which is very close to the emission wavelength of dye MoB-M in solid state (Figure S5). This result can further prove the formation of aggregates in water. As aforementioned, the aggregation process occurred in water solutions of dyes MeB-M and MoB as well. Therefore, we analyzed the normalized fluorescence emission spectra of dyes MeB-M and MoB (Figure 2B,C), finding two neglected shoulder peaks at longer wavelengths which are almost the same as their solid emission wavelength, respectively. Such a finding is highly consistent with our previous analysis.

The absorption maxima of dyes PyB and PyB-M in different solvents ranged from 455 to 487 nm and 456 to 494 nm, respectively (Figure S2E,F). Due to the poor solubility in water, dye PyB exhibited a weak and broad absorption spectrum, which is shown in Figure S1E. The fluorescence emission spectra of dyes PyB and PyB-M are summarized in Figure 2E,F. The emission maxima of PyB and PyB-M in different solvents were recorded to range from 503 to 654 nm and 501 to 559 nm. It is noteworthy that water caused an arresting emission red-shift of PyB to 654 nm, which can be ascribed to the fast and intense aggregation process.

For dyes EtB, EtB-M, EtHB, and EtHB-M, their absorption maxima in different solvents were in the ranges of 493~534 nm, 510~543 nm, 496~529 nm, and 502~541 nm, respectively (Figure S2G,J). As other dyes we discussed before, water can induce the rise of a side peak at shorter wavelength region, due to the solubility factor. After the introduction of hydroxy group, the solubility of dyes EtHB, and EtHB-M got worse in all the solvents we used. The fluorescence emission spectra of dyes EtB, EtB-M, EtHB, and EtHB-M are shown in Figure 2G–J. To be specific, their emission maxima in different solvents were recorded to range from 493 to 534 nm, 510 to 543 nm, 496 to 529 nm, and 502 to 541 nm, respectively. As a result of relatively good solubility, no obvious red-shift was observed in water and all other solvents applied.

Subsequently, we quantitatively analyzed the influence of solvent polarity on the Stokes shift (Δ*λ*) of all the BA-based dyes (Figure S4). Solvent polarity parameter Δ*f* was calculated by using the Lippert–Mataga equation [27]. According to the plots of Δ*f* vs. Δ*λ*, the Stokes shifts of EtHB and EtHB-M were mostly proportional to solvent polarities, suggesting they possess positive solvatochromism, like most of the fluorophores with push–pull structural feature. For all the other dyes, we observed that the Stokes shifts fluctuated in different solvents, possibly due to the aggregation issue that shed the dye molecules away from solvents that affect intramolecular charge transfer in the excited state. 

#### 2.2.3. Aggregation-Induced Emission

**Figure 3:**
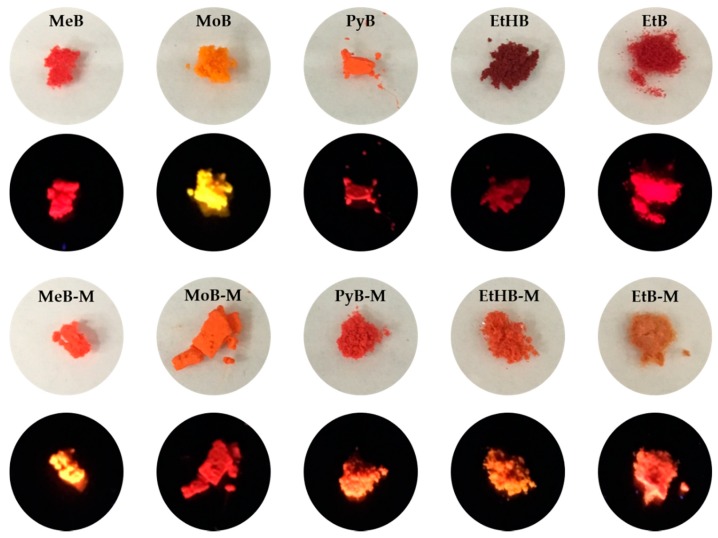
Photos of dyes MeB, MeB-M, MoB, MoB-M, PyB, PyB-M, EtHB, EtHB-M, EtB, and EtB-M in solid powder state under day light (upper line) and 365 nm UV light (lower line).

After synthesis of these BA-based compounds, we found that most of them emitted strong orange-to-red fluorescence in the solid state (Figure 3). As a contrast, they were only weakly emissive when molecularly dissolved in DMSO, a relatively good solvent for the BA-based compounds. The absolute quantum yields of all dyes in the solid state are shown in Table 1. In general, the methylated BA series exhibited higher quantum efficiency than the non-substituted counterparts, for example, 10.6% for MeB vs. 29.2% for MeB-M. The EtHB series has a lower solid-state quantum yield among all the compounds, consistent with the other results in solution. Therefore, these BA-based luminogens possess the unique AIE property.

**Figure 4:**
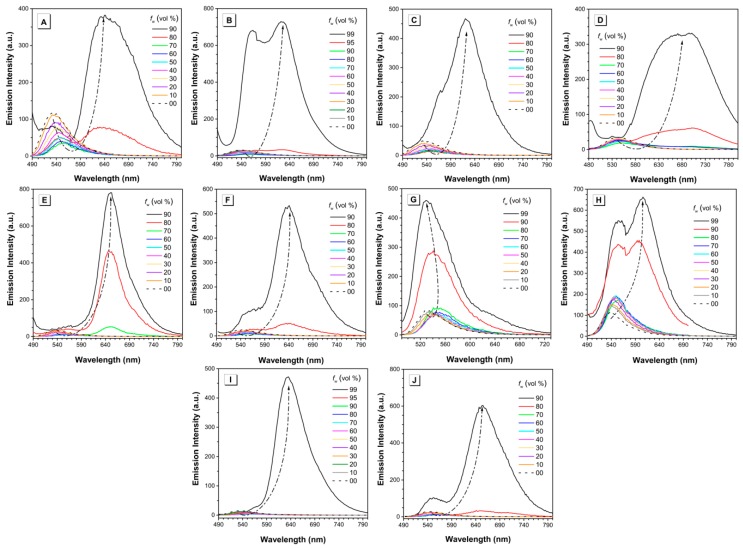
Emission intensity of (**A**) MeB, (**B**) MeB-M, (**C**) MoB, (**D**) MoB-M, (**E**) PyB, (**F**) PyB-M, (**G**) EtHB, (**H**) EtHB-M, (**I**) EtB, and (**J**) EtB-M in DMSO/water mixtures with different water fractions. Concentration = 100 µM. *f*_w_ = water fraction. *λ*_ex_ = 464, 468, 455, 459, 469, 473, 470, 474, 469, and 473 nm respectively.

**Figure 5:**
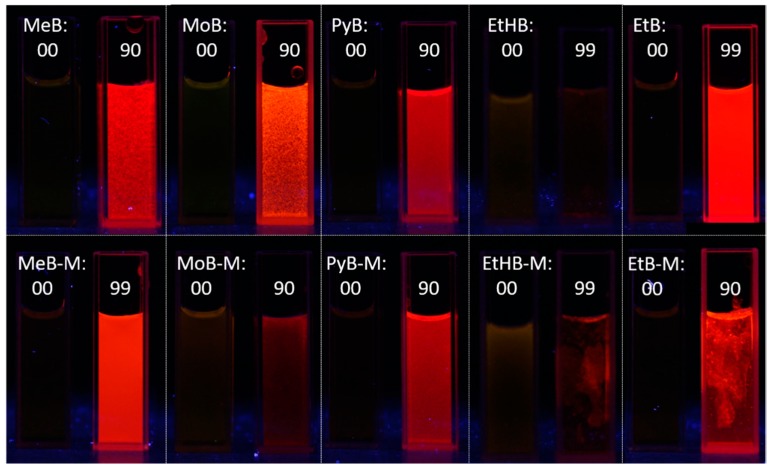
Fluorescence photos of dyes MeB, MeB-M, MoB, MoB-M, PyB, PyB-M, EtHB, EtHB-M, EtB, and EtB-M in solution (DMSO or methanol) and aggregation (*f*_w_ = 90 or 99%) state taken under a hand-held 365 nm UV lamp.

The AIE properties of all the BA-based dyes were investigated in a series of DMSO/water or methanol/water mixtures with different water fractions. Taking dye MeB as an example, it exhibited weak emission peaked at 530 nm when molecularly dissolved in DMSO (Figure 4A). With the addition of water, which acts as a poor solvent herein, the peak at 530 nm decreased gradually and shifted toward longer wavelength slightly. This is reasonable as the polarity of the mixed solution increased with the addition of water. When the water fraction reached 80%, a new peak emerged with the maximum located at ~640 nm. Such emission is similar to that of MeB in solid state. The fluorescence intensity reached the highest value when water fraction was 90%, showing the typical AIE phenomenon. Moreover, as shown in Figure 4, dyes MeB-M, MoB, MoB-M, PyB, PyB-M, EtB, and EtB-M showed similar behavior as dye MeB: they were wea or non-emissive until the water percentage increased up to 90%; thereafter, they showed a sharp increase in intensity. As an exception, both dyes EtHB and EtHB-M were non-emissive in the DMSO/water mixture even with 99% water fraction due to the poor solubility in the solvent system. Therefore, we optimized the solvent system and determined to use methanol/water pair to measure their AIE behavior. Finally, the fluorescence enhancement process can be observed with the gradual addition of water. As shown in Figure 5, the photos of all the dyes in solution and aggregation state were taken under UV light, which exhibited the AIE process intuitively. In summary, all these BA-based dyes are AIE-active luminogens which can be used in solid state or under high concentration. 

### 2.3. Application for Amyloid Protein Detection

**Figure 6:**
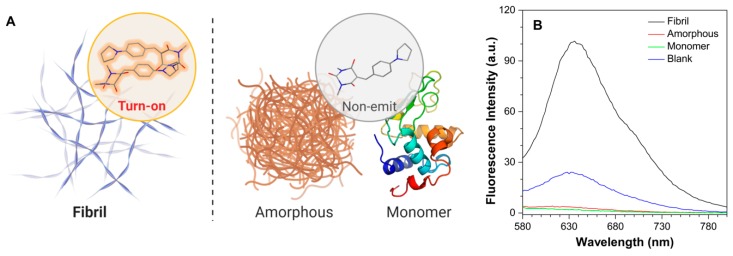
(**A**) Schematic diagrams showing the fluorescent turn-on effect of PyB-M toward protein fibrils. (**B**) Fluorescence emission spectra of PyB-M binding with fibril, amorphous, or monomer formations of HEWL. PyB-M concentration: 50 μM. *λ*_ex_ = 494 nm.

At the molecular level, highly ordered protein aggregates, termed amyloid fibrils, have been found to appear in a board range of neurodegenerative diseases before clinical symptoms manifest. Thus, protein fibrils have been regarded as a potential pre-symptomatically diagnostic biomarker, and recent efforts by many groups have demonstrated and expanded the utility of amyloid-targeting fluorophores as clinical tools for disease diagnostics [28,29]. AIE-based fluorogens, owing to their twisted molecular configurations and excellent photophysical properties, have been applied in recognition and differentiation of fibril-forming processes [30]. Moreover, it has been found that the coplanar geometry of a probe is crucial for fibril binding [22]. As such, we further employed dyes constructed by the planar BA moieties in the biological evaluation of fibril binding (Figure 6A). The fibril and amorphous formation of hen egg white lysozyme (HEWL) were prepared in acidic and alkaline pH, respectively, according to the previous publications [31,32]. As the counterpart, monomer solution of HEWL was made by directly dissolving powder in the acidic buffer. As shown in Figure 6B, PyB-M was slightly emitted in the water containing 1% DMSO (blank) and barely fluorescent in the HEWL amorphous and monomer solution. This phenomenon was ascribed to the poor solubility induced aggregates in water, whereas the increased hydrophobicity of protein solutions further extinct the emission of PyB-M. However, upon interacting with HEWL fibrils, the fluorescent intensity of PyB-M was markedly turned on, exhibiting 30-fold and 55-fold increases compared with HEWL amorphous and monomer solution, respectively. In contrast, as the routinely used amyloid detecting gold standard, thioflavin T (ThT) fluorescence showed no response in distinguishing HEWL fibrils and monomers (Figure S6). The protonated effect even triggered the ThT fluorescence in the blank condition. The finding is consistent with our previous observation that ThT cannot exert its function in a low-pH environment [30]. In this regard, PyB-M could be a better alternative to ThT, owing to its stability in low pH and emission at long wavelength (>600 nm) that can overcome the interference of autofluorescence from biological samples. 

## 3. Materials and Methods 

### 3.1. Materials and General Information

Barbituric acid, 1,3-dimethylbarbituric acid and 4-(diethylamino)benzaldehyde were purchased from Acros Organics (Geel, Belgium). 4-(Dimethylamino)benzaldehyde and DMSO was purchased from Sigma-Aldrich (Castle Hill, NSW, Australia). 4-(4-Morpholinyl)benzaldehyde, 4-(1-pyrrolidino)benzaldehyde and 4-(diethylamino)salicylaldehyde were purchased from Accela Chembio (Shanghai, China). Other solvents were purchased from Chem-supply (Adelaide, SA, Australia). All chemicals were used as received without further purification. 

^1^H-NMR and ^13^C-NMR spectra were recorded by using a 400 MHz Bruker AV3HD-400 spectrometer (Billerica, MA, USA) at 298 K, with tetramethylsilane (TMS, δ = 0) as the internal standard. HRMS spectra were acquired by using a Thermo Scientific Q Exactive Plus Orbitrap LC–MS/MS instrument operating in ESI (Electrospray ionization) mode. UV-visible absorption spectra were recorded at room temperature on a Cary 300 UV-visible spectrophotometer (Agilent Technologies, Inc., Santa Clara, CA, USA) equipped with a 1.0 cm quartz cell. Fluorescence emission spectra were recorded on a Cary Eclipse Fluorescence Spectrophotometer (Agilent Technologies, Inc., Santa Clara, CA, USA) and used 1.0 cm quartz cells. Data were plotted by using Origin 2019.

### 3.2. Synthesis and Characterization

The general synthetic method of BA-based dyes can be briefly described as follows: aldehyde compound (1.1 mmol) was added into a 100 mL round-bottom flask and dissolved with 8 mL of ethanol. Subsequently, equivalent molarity of (substituted) barbituric acid (1 mmol) in 2 mL of hot water was added, and the mixture was heated up to 60 °C and stirred for about 6 h. After cooling, the resulting solid was collected, washed three times with ice-cold ethanol, and dried over, to afford the final product.

5-(4-(dimethylamino)benzylidene)pyrimidine-2,4,6(1H,3H,5H)-trione (**MeB**) (0.23 g, yield 89%). ^1^H-NMR (400 MHz, DMSO-d_6_): δ (ppm): 10.95 (s, 2H), 8.42 (d, J = 9.2 Hz, 2H), 8.15 (s, 1H), 6.80 (d, J = 9.2 Hz, 2H), 3.12 (s, 6H). ^13^C-NMR (101 MHz, DMSO-d_6_): δ (ppm): 165.15, 163.18, 155.93, 154.63, 150.76, 139.50, 120.45, 111.66, 110.02. HRMS (ESI-MS): m/z 260.1023, calcd. 259.0957.

5-(4-(dimethylamino)benzylidene)-1,3-dimethylpyrimidine-2,4,6(1H,3H,5H)-trione (**MeB-M**) (0.26 g, yield 92%). ^1^H-NMR (400 MHz, DMSO-d_6_): δ (ppm): 8.41 (d, J = 9.3 Hz, 2H), 8.24 (s, 1H), 6.83 (d, J = 9.3 Hz, 2H), 3.23 (s, 6H), 3.14 (s, 6H). ^13^C-NMR (101 MHz, DMSO-d_6_): δ (ppm): 163.71, 161.66, 156.79, 154.72, 151.72, 139.57, 120.50, 111.67, 109.80, 28.98, 28.35. HRMS (ESI-MS): m/z 288.1342, calcd. 287.1270.

5-(4-morpholinobenzylidene)pyrimidine-2,4,6(1H,3H,5H)-trione (**MoB**) (0.23 g, yield 75%). ^1^H-NMR (400 MHz, DMSO-d_6_): δ (ppm): 11.06 (d, J = 50.6 Hz, 2H), 8.39 (d, J = 9.1 Hz, 2H), 8.16 (s, 1H), 7.02 (d, J = 9.2 Hz, 2H), 3.77–3.69 (m, 4H), 3.50–3.43 (m, 4H). ^13^C-NMR (101 MHz, DMSO-d_6_): δ (ppm): 164.91, 163.02, 155.66, 154.66, 150.72, 138.99, 122.12, 112.88, 111.97, 66.25, 46.66. HRMS (ESI-MS): m/z 302.1143, calcd. 301.1063.

1,3-dimethyl-5-(4-morpholinobenzylidene)pyrimidine-2,4,6(1H,3H,5H)-trione (**MoB-M**) (0.26 g, yield 80%). ^1^H-NMR (400 MHz, DMSO-d_6_): δ (ppm): 8.41 (d, J = 9.3 Hz, 2H), 8.26 (s, 1H), 7.05 (d, J = 9.3 Hz, 2H), 3.73 (m, 4H), 3.48 (m, 4H), 3.23 (s, 6H). HRMS (ESI-MS): m/z 330.1446, calcd. 329.1376.

5-(4-(pyrrolidin-1-yl)benzylidene)pyrimidine-2,4,6(1H,3H,5H)-trione (**PyB**) (0.27 g, yield 95%). ^1^H-NMR (400 MHz, DMSO-d_6_): δ (ppm): 10.94 (d, J = 49.0 Hz, 2H), 8.44 (d, J = 9.1 Hz, 2H), 8.14 (s, 1H), 6.66 (d, J = 9.2 Hz, 2H), 3.44 (t, J = 6.5 Hz, 4H), 1.99 (t, J = 6.6 Hz, 4H). ^13^C-NMR (101 MHz, DMSO-d_6_): δ (ppm): 165.22, 163.23, 156.00, 152.18, 150.78, 139.79, 120.39, 112.20, 109.37, 48.11, 25.30. HRMS (ESI-MS): m/z 286.1182, calcd. 285.1113.

1,3-dimethyl-5-(4-(pyrrolidin-1-yl)benzylidene)pyrimidine-2,4,6(1H,3H,5H)-trione (**PyB-M**) (0.29 g, yield 93%). ^1^H-NMR (400 MHz, DMSO-d_6_): δ (ppm): 8.44 (d, J = 9.1 Hz, 2H), 8.24 (s, 1H), 6.68 (d, J = 9.2 Hz, 2H), 3.46 (m, 4H), 3.23 (s, 6H), 2.00 (s, 4H). ^13^C-NMR (101 MHz, DMSO-d_6_): δ (ppm): 163.76, 161.69, 156.87, 152.28, 151.74, 139.86, 120.45, 112.24, 109.15, 48.15, 28.96, 28.34, 25.30. HRMS (ESI-MS): m/z 314.1494, calcd. 313.1426.

5-(4-(diethylamino)-2-hydroxybenzylidene)pyrimidine-2,4,6(1H,3H,5H)-trione (**EtHB**) (0.18 g, yield 58%). ^1^H-NMR (400 MHz, DMSO-d_6_): δ (ppm): 10.77 (s, 1H), 8.99 (d, J = 9.5 Hz, 1H), 8.76 (s, 1H), 6.35 (dd, J = 9.6, 2.4 Hz, 1H), 6.16 (d, J = 2.5 Hz, 1H), 3.44 (q, J = 7.0 Hz, 4H), 1.15 (t, J = 7.0 Hz, 6H). ^13^C-NMR (101 MHz, DMSO-d_6_): δ (ppm): 164.43, 155.32, 150.90, 148.80, 137.29, 110.33, 106.59, 105.25, 95.89, 44.84, 13.13. HRMS (ESI-MS): m/z 304.1289, calcd. 303.1219.

5-(4-(diethylamino)-2-hydroxybenzylidene)-1,3-dimethylpyrimidine-2,4,6(1H,3H,5H)-trione (**EtHB-M**) (0.14 g, yield 43%). ^1^H-NMR (400 MHz, DMSO-d_6_): δ (ppm): 10.69 (s, 1H), 8.94 (d, J = 9.6 Hz, 1H), 8.85 (s, 1H), 6.38 (d, J = 9.6 Hz, 1H), 6.17 (d, J = 2.5 Hz, 1H), 3.45 (m, 4H), 3.21 (S, 6H), 1.16 (m, 6H). ^13^C-NMR (101 MHz, DMSO-d_6_): δ (ppm): 164.54, 164.13, 161.81, 155.42, 151.86, 149.79, 137.09, 110.48, 106.38, 105.28, 95.83, 44.88, 28.82, 28.24, 13.13. HRMS (ESI-MS): m/z 332.1608, calcd. 331.1532.

5-(4-(diethylamino)benzylidene)pyrimidine-2,4,6(1H,3H,5H)-trione (**EtB**) (0.23 g, yield 81%). ^1^H-NMR (400 MHz, DMSO-d_6_): δ (ppm): 10.95 (d, J = 38.5 Hz, 2H), 8.41 (d, J = 9.3 Hz, 2H), 8.12 (s, 1H), 6.79 (d, J = 9.3 Hz, 2H), 3.51 (q, J = 7.0 Hz, 4H), 1.16 (t, J = 7.0 Hz, 6H). ^13^C-NMR (101 MHz, DMSO-d_6_): δ (ppm): 165.23, 163.21, 155.71, 152.71, 150.77, 139.98, 120.13, 111.39, 109.46, 44.71, 12.96. HRMS (ESI-MS): m/z 288.1344, calcd. 287.1270.

5-(4-(diethylamino)benzylidene)-1,3-dimethylpyrimidine-2,4,6(1H,3H,5H)-trione (**EtB-M**) (0.24 g, yield 77%). ^1^H-NMR (400 MHz, DMSO-d_6_): δ (ppm): 8.41 (d, J = 9.3 Hz, 2H), 8.21 (s, 1H), 6.80 (d, J = 9.3 Hz, 2H), 3.52 (q, J = 7.0 Hz, 4H), 3.22 (s, 6H), 1.16 (t, J = 7.0 Hz, 6H). ^13^C-NMR (101 MHz, DMSO-d_6_): δ (ppm): 163.75, 161.66, 156.59, 152.82, 151.72, 140.04, 120.18, 111.40, 109.21, 44.74, 28.95, 28.32, 12.96. HRMS (ESI-MS): m/z 316.1652, calcd. 315.1583.

### 3.3. Sample Preparation for Spectroscopy Measurement

For solvatochromism experiments, all stock solutions of dyes were prepared at 1 mM in DMSO. Afterward, the stock solutions were diluted 100 times, using different solvents, to obtain working solutions with a concentration of 10 μM. In UV-vis absorption measurement, the background of solvent alone was subtracted. For the AIE curve measurement, a stock solution of BA-based dye in DMSO (1 or 10 mM) was first prepared. Aliquots of this stock solution were transferred into volumetric flasks (10 mL), into which appropriate volumes of DMSO and water were added dropwise, under vigorous stirring, to furnish 100 µM solutions with different water contents (0−99 vol%). Fluorescence emission spectra were measured immediately after the solutions were prepared.

### 3.4. Quantum Yield Measurements

Solid film fluorescence quantum yields were measured, using a Hamamatsu absolute PL quantum yield spectrometer C11347 Quantaurus_QY (Hamamatsu City, Shizuoka Prefecture, Japan) and a matched integrating sphere. Data showed averaged values of three detected readings from the machine. 

### 3.5. Protein-Formation Detection

The preparation methods of protein samples were adopted from the strategies previously described by Carver et al. [30], which are summarized in detail in Table 2. 

The dye PyB-M was prepared at 5 mM in DMSO, as the stock solution. Assays were conducted by 1:100 diluting the stock solution of dyes into protein samples, and emission spectra were immediately recorded by the fluorescence spectrophotometer. The excitation wavelengths of PyB-M and ThT were set at 494 and 440 nm, respectively. 

## 4. Conclusions

In summary, we synthesized ten organic AIE-active fluorescent dyes through the simple and robust Knoevenagel condensation reaction of BA blocks and benzaldehydes. Among these dyes, PyB, PyB-M, MoB-M, and EtHB-M are new molecules, which have never been reported before. All these BA-based dyes exhibited long-wavelength absorption and emission in the orange-to-red region. Besides, most of the dyes in solid state enjoy high quantum yields, which can be as high as 32.8%. Thanks to the electron pull–push architecture, these dyes exhibited the special photophysical property of solvatochromism. Finally, the AIE behaviors of these dyes were demonstrated by using standard fluorometric techniques. We further demonstrated the application of differentiating pathogenic amyloid fibrils from other protein conformers by using one of the novel analogues. Further application of the BA-based dyes in biosensing is ongoing in our lab. 

## Figures and Tables

**Table 1:** Photophysical properties of the BA-based dyes.

Compound	Solution Absorbance λ_max_ (nm) ^[1]^	Molar Absorptivity at λ_max_ (M^−^^1^ cm^−1^) ^[1]^	Solution Emission λ_max_ (nm) ^[1]^	Solid Emission λ_max_ (nm)	Quantum Yield of Solid Film (%)
**MeB**	464	6.58 × 10^4^	526	629	10.6
**MeB-M**	468	6.06 × 10^4^	538	595	29.2
**MoB**	455	4.90 × 10^4^	527	589	15.5
**MoB-M**	459	5.22 × 10^4^	537	621	\ ^[2]^
**PyB**	469	7.02 × 10^4^	527	640	9.0
**PyB-M**	473	6.54 × 10^4^	536	616	14.1
**EtHB**	471	6.84 × 10^4^	521	644	1.5
**EtHB-M**	474	1.09 × 10^5^	530	603	2.5
**EtB**	469	5.76 × 10^4^	532	638	19.1
**EtB-M**	473	6.63 × 10^4^	541	656	32.8

^1^ Measured in DMSO solutions. ^2^ Data cannot be obtained due to poor solubility and film-forming property.

**Table 2:** Aggregation conditions for HEWL and the corresponding aggregate types.

Concentration	Aggregation Condition	Type of Aggregate
35 µM	50 mM of glycine-HCl pH 2.0, 137 mM of NaCl, 2.7 mM of KCl, 0.01% (w/v) NaN_3_, 55 °C, medium shaking, 8 h [31]	Fibril
70 µM	50 mM of glycine-HCl pH 12.2, 37 °C, 300 rpm, 8 h [32]	Amorphous
70 µM	50 mM of glycine-HCl pH 2.0	Monomer

## Supplementary Materials

Supplementary materials are available online.
