# Supplementary material for: Barbituric Acid Based Fluorogens: Synthesis, Aggregation-Induced Emission, and Protein Fibril Detection
Source: Molecules. 2019 Dec 20;25(1):32. doi: 10.3390/molecules25010032 (PMC6982923; doi:10.3390/molecules25010032)
Supplement: Supplementary file 1 [file molecules-25-00032-s001.pdf]

Supplementary Information

Barbituric Acid Based Fluorogens: Synthesis, Aggregation-Induced Emission and Protein Fibril Detection

Siyang Ding [1,#], Bicheng Yao [1,#], Louis Schobben [1], and Yuning Hong [1,*]

[1] Department of Chemistry and Physics, La Trobe Institute for Molecular Science, La Trobe University, Melbourne, VIC 3086, Australia; Siyang.Ding@latrobe.edu.au (S.D.); b.yao@latrobe.edu.au (B.Y.); 19341287@students.latrobe.edu.au (L.S.); y.hong@latrobe.edu.au (Y.H.)

[#] Equal contribution

* Correspondence: y.hong@latrobe.edu.au; Tel.: +61-3-9479-2995 (Y.H.)

Table of Contents

Figure S1. UV-vis absorption spectra of dyes (A) **MeB**, (B) **MeB-M**, (C) **MoB**, (D) **MoB-M**, (E) **PyB**, (F) **PyB-M**, (G) **EtHB**, (H) **EtHB-M**, (I) **EtB** and (J) **EtB-M** in different solvents. 2

Figure S2. Normalized absorption spectra of dyes (A) **MeB**, (B) **MeB-M**, (C) **MoB**, (D) **MoB-M**, (E) **PyB**, (F) **PyB-M**, (G) **EtHB**, (H) **EtHB-M**, (I) **EtB** and (J) **EtB-M** in different solvents. 2

Figure S3. Fluorescence emission spectra of dyes (A) **MeB**, (B) **MeB-M**, (C) **MoB**, (D) **MoB-M**, (E) **PyB**, (F) **PyB-M**, (G) **EtHB**, (H) **EtHB-M**, (I) **EtB** and (J) **EtB-M** in different solvents. 3

Figure S4. Lippert–Mataga solvatochromism plot of dyes (A) **MeB**, (B) **MeB-M**, (C) **MoB**, (D) **MoB-M**, (E) **PyB**, (F) **PyB-M**, (G) **EtHB**, (H) **EtHB-M**, (I) **EtB** and (J) **EtB-M** in different solvents as a function of solvent polarity measured by Lippert-Mataga polarity parameter. 3

Figure S5. Normalized fluorescence emission spectra of dyes **MeB**, **MeB-M**, **MoB**, **MoB-M**, **PyB**, **PyB-M**, **EtHB**, **EtHB-M**, **EtB** and **EtB-M** in solid powder state. 4

Figure S6. Fluorescence emission spectra of ThT binding with fibril, amorphous or monomer formations of HEWL. 4

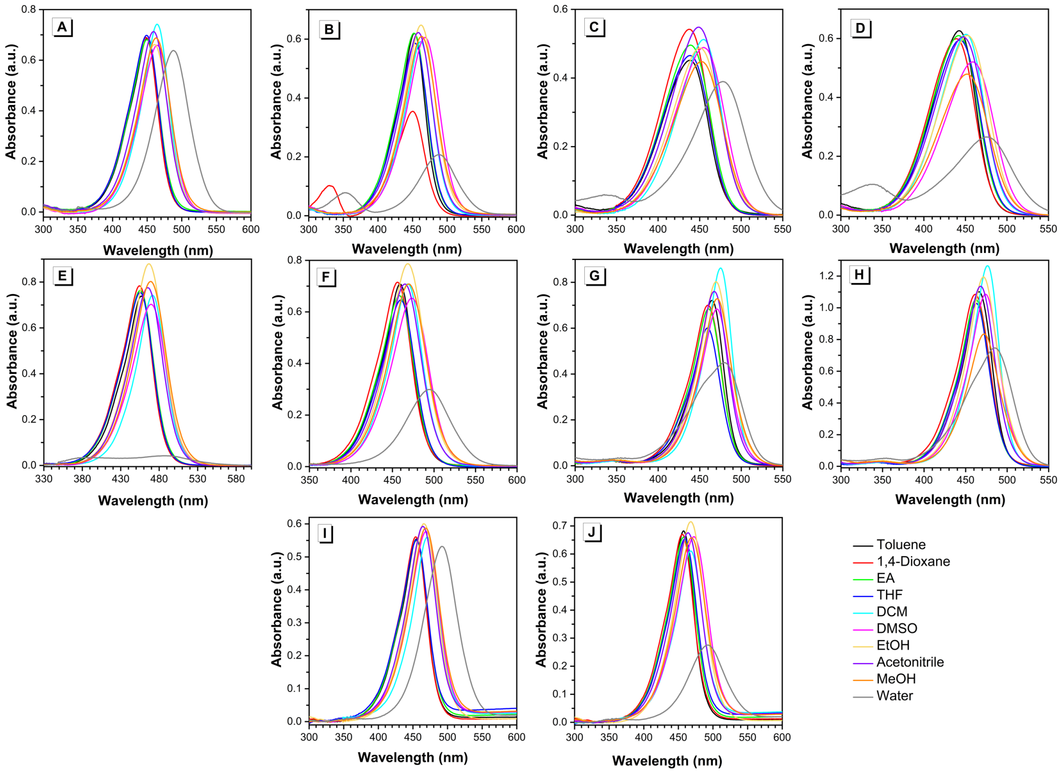

Figure S1. UV-vis absorption spectra of dyes (A) **MeB**, (B) **MeB-M**, (C) **MoB**, (D) **MoB-M**, (E) **PyB**, (F) **PyB-M**, (G) **EtHB**, (H) **EtHB-M**, (I) **EtB** and (J) **EtB-M** in different solvents. Concentration = 10 μM.

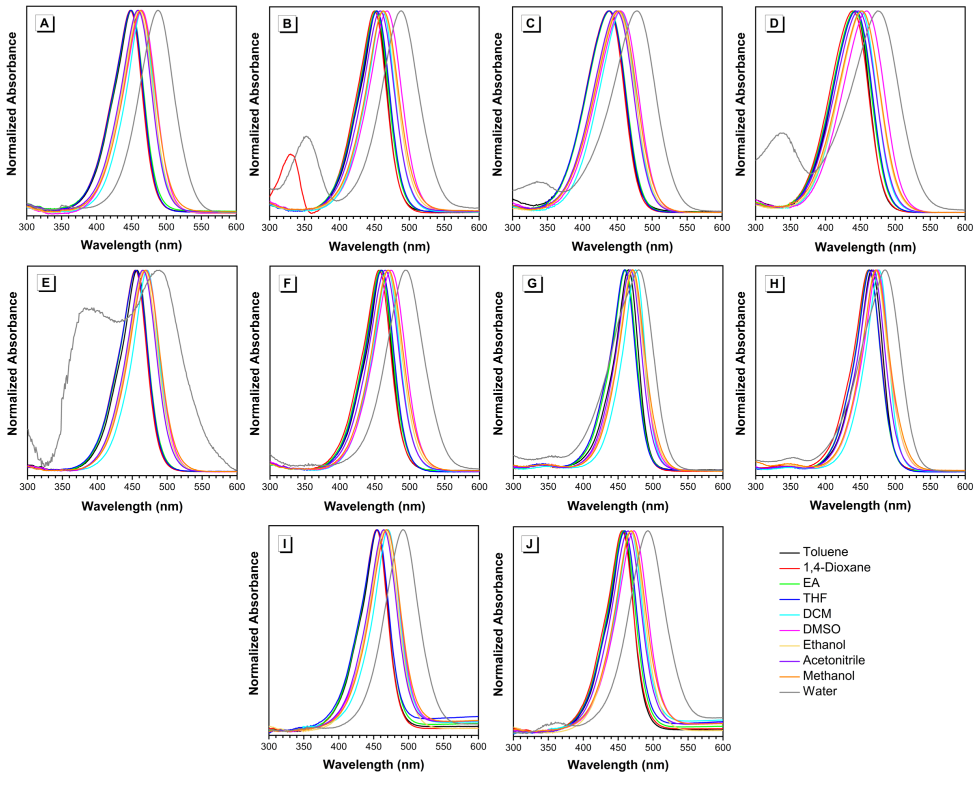

Figure S2. Normalized absorption spectra of dyes (A) **MeB**, (B) **MeB-M**, (C) **MoB**, (D) **MoB-M**, (E) **PyB**, (F) **PyB-M**, (G) **EtHB**, (H) **EtHB-M**, (I) **EtB** and (J) **EtB-M** in different solvents. Concentration = 10 μM.

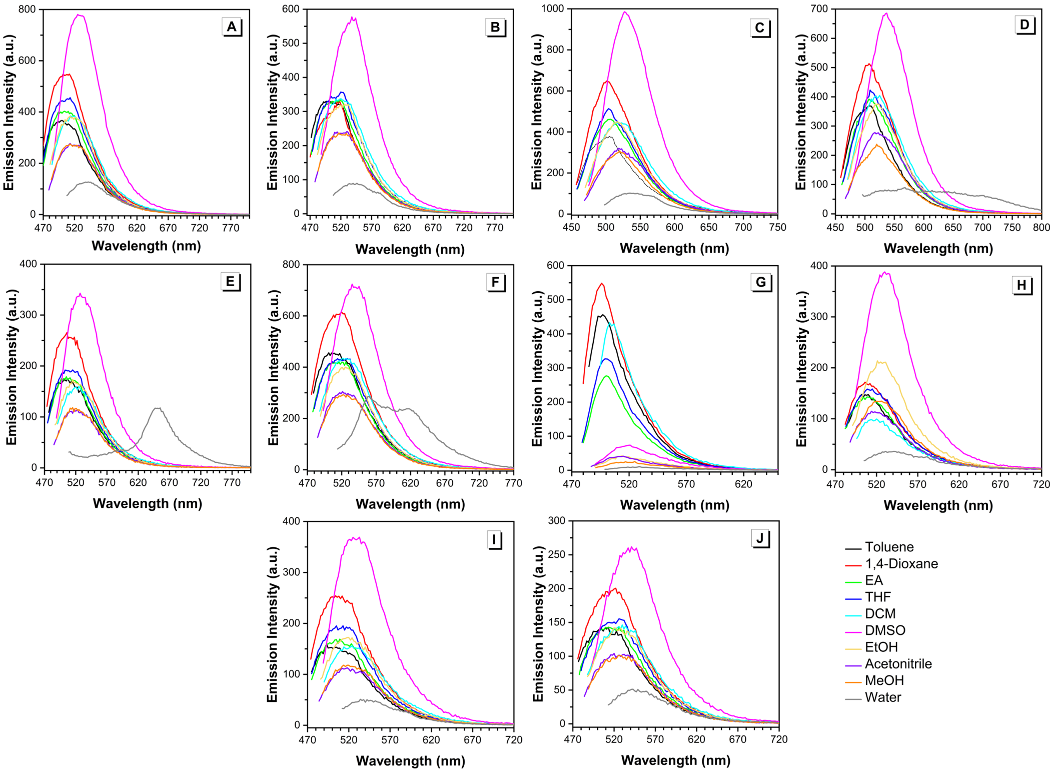

Figure S3. Fluorescence emission spectra of dyes (A) **MeB**, (B) **MeB-M**, (C) **MoB**, (D) **MoB-M**, (E) **PyB**, (F) **PyB-M**, (G) **EtHB**, (H) **EtHB-M**, (I) **EtB** and (J) **EtB-M** in different solvents. Concentration = 10 μM. λ_{ex} = 438~494 nm.

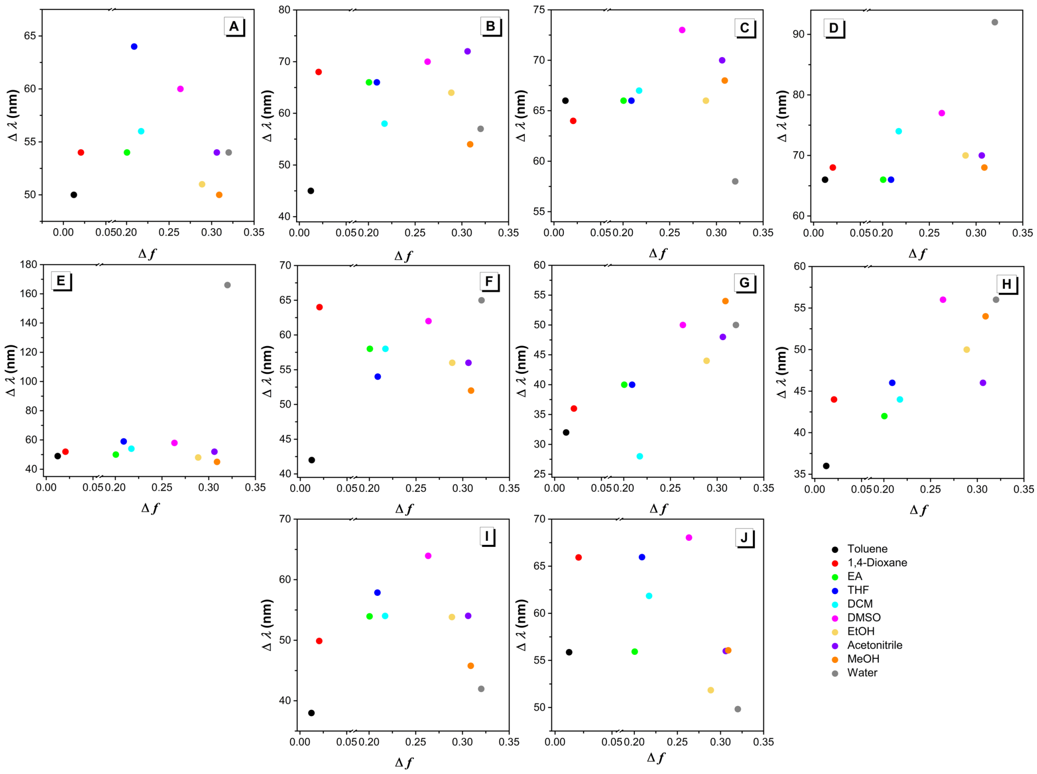

Figure S4. Lippert–Mataga solvatochromism plot of dyes (A) **MeB**, (B) **MeB-M**, (C) **MoB**, (D) **MoB-M**, (E) **PyB**, (F) **PyB-M**, (G) **EtHB**, (H) **EtHB-M**, (I) **EtB** and (J) **EtB-M** in different solvents as a function of solvent polarity measured by Lippert-Mataga polarity parameter.

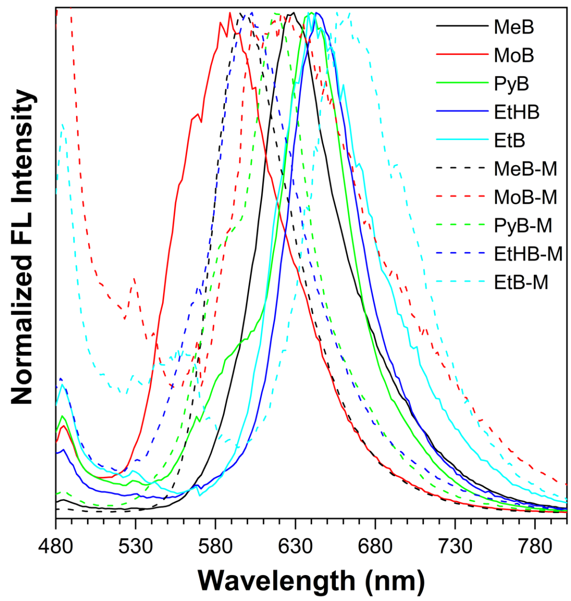

Figure S5. Normalized fluorescence emission spectra of dyes **MeB**, **MeB-M**, **MoB**, **MoB-M**, **PyB**, **PyB-M**, **EtHB**, **EtHB-M**, **EtB** and **EtB-M** in solid powder state. λ_{ex} = 438~462 nm.

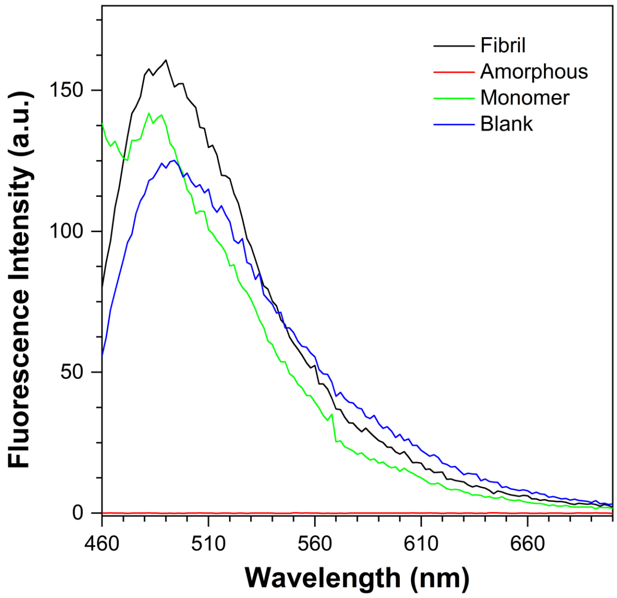

Figure S6. Fluorescence emission spectra of ThT binding with fibril, amorphous or monomer formations of HEWL. ThT concentration: 50 μM. λ_{ex} = 440 nm.
